# Cardiovascular function, compliance, and connective tissue remodeling in the turtle, *Trachemys scripta*, following thermal acclimation

**DOI:** 10.1152/ajpregu.00510.2015

**Published:** 2016-04-13

**Authors:** Adam N. Keen, Holly A. Shiels, Dane A. Crossley

**Affiliations:** ^1^Faculty of Life Sciences, University of Manchester, Manchester, United Kingdom; and; ^2^Department of Biological Sciences, University of North Texas, Denton, Texas

**Keywords:** heart, in vivo, blood flow, blood pressure, cardiac preload, stiffness, collagen, elastin

## Abstract

Low temperature directly alters cardiovascular physiology in freshwater turtles, causing bradycardia, arterial hypotension, and a reduction in systemic blood pressure. At the same time, blood viscosity and systemic resistance increase, as does sensitivity to cardiac preload (e.g., via the Frank-Starling response). However, the long-term effects of these seasonal responses on the cardiovascular system are unclear. We acclimated red-eared slider turtles to a control temperature (25°C) or to chronic cold (5°C). To differentiate the direct effects of temperature from a cold-induced remodeling response, all measurements were conducted at the control temperature (25°C). In anesthetized turtles, cold acclimation reduced systemic resistance by 1.8-fold and increased systemic blood flow by 1.4-fold, resulting in a 2.3-fold higher right to left (R-L; net systemic) cardiac shunt flow and a 1.8-fold greater shunt fraction. Following a volume load by bolus injection of saline (calculated to increase stroke volume by 5-fold, ∼2.2% of total blood volume), systemic resistance was reduced while pulmonary blood flow and systemic pressure increased. An increased systemic blood flow meant the R-L cardiac shunt was further pronounced. In the isolated ventricle, passive stiffness was increased following cold acclimation with 4.2-fold greater collagen deposition in the myocardium. Histological sections of the major outflow arteries revealed a 1.4-fold higher elastin content in cold-acclimated animals. These results suggest that cold acclimation alters cardiac shunting patterns with an increased R-L shunt flow, achieved through reducing systemic resistance and increasing systemic blood flow. Furthermore, our data suggests that cold-induced cardiac remodeling may reduce the stress of high cardiac preload by increasing compliance of the vasculature and decreasing compliance of the ventricle. Together, these responses could compensate for reduced systolic function at low temperatures in the slider turtle.

reductions in ambient temperature have direct (i.e., Q_10_; the rate of change over 10°C) and immediate effects on the ectotherm heart and cardiovascular system. This response to low temperature is clear in freshwater turtles, resulting in decreased heart rate (*f*_H_), cardiac twitch force, cardiac output (Q_total_), and ventricular power output (15, 27). However, cardiac stroke volume (VS_tot_) is maintained at low temperatures, partly by reduced end-systolic volume and increased diastolic filling due to changes in vascular resistance, and the sensitivity of the turtle heart to cardiac preload is increased (15, 27). These temperature-induced responses and the direct effect of temperature on blood viscosity may alter hemodynamic load on the heart (44, 56, 62). If prolonged, these physical and functional changes may trigger a dynamic remodeling of the passive and active properties of the cardiovascular system (62, 63).

Freshwater slider turtles (*Tracheyms scripta*) spend winter in water in a state of periodic inactivity. Following cold acclimation slider turtles exhibit a compensatory increase in cardiac muscle twitch force and maximal isometric force, as well as a suppression of cholinergic inhibition, slower action potential upstroke, and longer action potential duration, accompanied by a dissipation of resting membrane potential (28, 51, 59, 62, 64). However, heart and ventricular mass do not increase (51). With the increased sensitivity to cardiac preload (15) and ability for a large Frank-Starling response (19), it has been suggested that VS_tot_ has the potential to increase fivefold in diving slider turtles (6).

Cardiac parameters of warm- and cold-acclimated turtles have been previously reported in a number of studies, which are in general agreement that following cold acclimation *f*_H_, Q_total_, and P_sys_ are decreased while VS_tot_, systemic resistance (R_sys_) and hematocrit are increased (27, 51, 62, 64). However, these studies were performed at the turtle's acclimation temperature (i.e., ∼5 and 25°C for cold and control, respectively). While this experimental methodology allows determinations of cardiac function at the acclimation temperature, the physical changes to the cardiovascular system caused by cold acclimation are difficult to isolate from the direct effect of cold temperature. Studies in rainbow trout have shown significant structural remodeling of the cardiovascular system, which persists in the absence of the direct effects of low temperature (39, 42). Therefore, to differentiate a remodeling response from the direct effect of temperature, cardiovascular function must be assessed at a common “test” temperature.

Our objective was to determine whether prolonged temperature acclimation caused remodeling of the freshwater red-eared slider turtle (*Tracheyms scripta*) cardiovascular system. To differentiate the direct effect of temperature, and focus on the longer term remodeling response of the cardiovascular system, animals were acclimated to a cold (5 ± 0.3°C) or control temperature (25 ± 0.3°C), but all experiments were conducted at the control temperature (of 25 ± 0.3°C). We assessed in vivo cardiac parameters in anesthetized animals after a period of postsurgical stabilization and then in response to a bolus injection (mean = 2.2 ± 0.4% of total blood volume) of saline directly into the jugular vein. Our first hypothesis was that chronic cold would reduce Q_total_, increase VS_tot_, and decrease P_sys_. Our second hypothesis was that cold acclimation would dampen the cardiac response to increased cardiac preload. As turtles have incomplete ventricular separation of venous and arterial circulations, allowing blood flow to bypass or partially bypass either systemic or pulmonary circulation (29, 30, 32), we were particularly interested in cardiac shunt flow (Q_shunt_) patterns. We further investigated the physical properties of the ventricle hypothesizing that cold acclimation would cause an increase in passive stiffness of the ventricle, which would be reflected in an increased collagen deposition. We found that Q_shunt_ patterns were altered following cold acclimation, with a right to left (R-L; net systemic) Q_shunt_ achieved by reducing R_sys_ and, therefore, increasing Q_sys_. Furthermore, it appears that cold-induced cardiovascular remodeling increases ventricular stiffness and systemic vasculature compliance, which reduces the stress of high blood volume load and may compensate for decreased systolic function at low temperatures.

## MATERIALS AND METHODS

### 

#### Experimental animals and acclimation.

Male and female red-eared sliders (*Trachemys scripta*, Schoepff; *n* = 20; mean body mass = 1,352 ± 69 g) were obtained from Lake Lewisville, TX, and transported to the University of North Texas. Here, they were housed in ∼50 L plastic containers (dimensions ∼50 × 50 × 100 cm) containing freshwater at a temperature of 25 ± 0.3°C on a 12:12-h light-dark cycle. Water quality was maintained by 100% water changes twice a week and all animals were fed three times per week on commercial reptile feed (Aquatic turtle diet, Mazuri exotic animal nutrition). After 2 wk, 10 turtles were randomly assigned for cold acclimation where ambient temperature was reduced by 1°C per day until 5 ± 0.3°C was reached and turtles were maintained at this temperature for 8–12 wk before experiments. An acclimation period of >8 wk was chosen to agree with our recent studies on fish (39), where >8 wk acclimation time is necessary to ensure cardiovascular structural remodeling (18, 42). Animals selected for cold acclimation were fasted following temperature reduction. The acclimation temperatures (control at 25 ± 0.3°C; cold at 5 ± 0.3°C) were chosen based on previous literature (27, 28) to simulate summer and winter conditions. All acclimation temperatures were maintained in a walk in temperature controlled room (model IR-912L5; Percival Scientific, Perry, IA) and animals were maintained in water without an area for basking. All animals survived the acclimation protocols and there were no signs of poor health in either group. Animal care and surgical preparations adhered to the University of North Texas animal care and use protocol (Institutional Animal Care and Use Committee No. 11-007).

#### Anesthesia and surgical procedure.

Before study, control animals were fasted for 1 wk. On the day of study, turtles were removed from acclimation tanks, immediately weighed, and then anesthetized via an intramuscular injection of sodium pentobarbital (50 mg/kg; Sigma-Aldrich, St. Louis, MO) while still at their acclimation temperature. The sodium pentobarbital was purchased as a powder and made as 2 ml 100% ethanol, 8 ml propylene glycol, and 10 ml saline with 1,000 mg pentobarbital. Animals were then transported to the experimental chamber (at 25 ± 0.3°C). In most cases the pedal withdrawal response ceased within 30–60 min postinjection; however, if it persisted, an additional injection (25 mg/kg) was administered. During surgery, and throughout experiments, turtles were maintained ventral side up and artificially ventilated to maintain normoxia via a tube inserted through the glottis into the trachea (model 665; Harvard Apparatus, Holliston, MA) at a rate of 24 breaths/min and a volume of 20 ml as previously reported for studies of this species (11, 13). Gas composition was controlled by rotameters (Sho-Rate Brooks Instruments Division, Hatfield, PA) and bubbled into a gas mixer to maintain hydration. Fractional CO_2_ (F_CO_2__) was maintained at 3 kPa to mimic partial pressure of CO_2_ in arterial blood (Pco_2_) (11, 22). The ventilated gas mixture (F_O_2__ = 0.21, F_CO_2__ = 0.03, balance N_2_) was checked regularly using a S-3A/I oxygen analyzer and a CD-3A carbon dioxide analyzer (Ametek, Berwyn, PA).

**Fig. 1.**
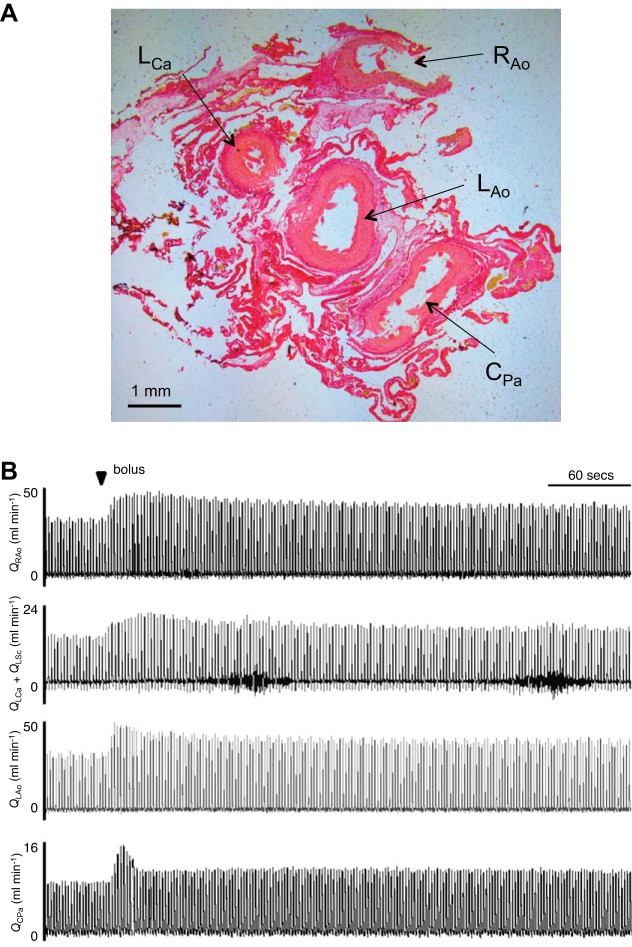
Flow probe placement. *A*: representative histological section through the outflow vessels directly above the turtle heart, stained with picro-sirus red, around which the flow probes were placed. Flow probes were placed around the common pulmonary artery (C_Pa_), the left aorta (L_Ao_), the left carotid artery (L_Ca_) and left subclavian artery (L_SCa_), and the right aortic bundle (R_Ao_). *B*: original representative recordings of blood flow in the right vessel bundle (Q_RAo_), accumulative left carotid and left subclavian (Q_LCa_ and Q_LSCa_), left aorta (Q_LAo_), and common pulmonary artery (Q_CPa_) taken from one anesthetized turtle immediately before and after exogenous volume load calculated to increase average stroke volume by 5-fold, via the external jugular vein. The arrow marks the point at which the volume load (bolus) was injected.

To expose the central vascular blood vessels, a 5 × 5 cm section of the plastron was cut away using a bone saw. The pectoral muscles and connective tissue were gently loosened from the excised piece and bleeding from small superficial vessels stopped by cauterization. For measurements of blood flows, 1- to 1.5-cm sections of all of the major outflow vessels of the heart were freed from connective tissue by blunt dissection, taking care not to damage any smaller branching vessels or perforate the pericardium. 2S transit-time ultrasonic blood flow probes (Transonic Systems, Ithica, NY) were fitted around all of the major outflow vessels. The first around the common pulmonary artery (C_Pa_), the second around the left aorta (L_Ao_), the third around both the left carotid and subclavian arteries (L_Ca_ and L_SCa_) and the final around the right aortic bundle (R_Ao_) (Fig. 1*A* shows the major vessels as a histological section) (69). After flow probe placement, a small incision was made in the left side of the neck and the left carotid artery and exterior jugular vein were exposed by blunt dissection. Both vessels were occlusively cannulated with polyethylene tubing (PE50) containing heparinized (50 units/ml) saline (0.9% NaCl) and the incision was sutured closed. Finally, a small incision was made in the pericardium to expose the apex of the heart. During each heartbeat, the separation of the cavum arteriosum and cavum venosum could be visualized. A small hole was made in the heart wall, into the cavum arteriosum, using a 28-gauge needle, and polyethylene tubing (PE10) containing heparinized saline was inserted into the heart. The same process was repeated for the cavum venosum. We are confident that this method allowed for correct cannula placement; however, due to the subsequent passive filling experiments we could not verify this post mortem. All catheters were connected to pressure transducers, which were calibrated daily against a static column of water by a two-point calibration at 0 and 1 kPa. Before experimental procedures animals were left for a minimum of 1-h stabilization period. After 1 h all pressures and flows were checked to ensure they had been stable for at least 30 min; if they were not, the animals were left until they had. The total time from anesthetic dose to commencing experiments was ∼4 h, during which cold-acclimated animals warmed to the control temperature.

#### In vivo cardiovascular measurements.

Each catheter was attached to a disposable pressure transducer (model MLT0699; ADInstruments, Colorado Springs, CO), adjusted to sit at the same level as the animal's heart, connected to an amplifier (Quad Bridge Amp; ADInstruments). Flow probes were connected to T206 dual channel small animal blood flow meters (Transonic Systems, Ithica, NY) for instantaneous blood flow rates. The output from the transonic meters and the pressure signal were acquired at 40 Hz using a PowerLab 16/35 data recording system (ADInstruments) and LabChart Pro software (v 7.2.5; ADInstruments).

All experiments were conducted at the control temperature of 25 ± 0.3°C. Turtle body temperature was monitored by a cloacal thermistor and a temperature probe (BAT-12 Microprobe Thermometer; Physitemp, Clifton, NJ) placed in the chest cavity to ensure it remained at 25 ± 0.3°C throughout experiments. The control temperature was chosen as the common temperature, instead of 5 ± 0.3°C, as there is a body of literature on turtle heart function under anesthesia at ∼25°C (11, 13, 36, 50). After stable pressures and flows were ensured, baseline recordings of all pressures and flows were taken for a 5-min period. During this time, average VS_tot_ was calculated for each individual. Following baseline recording, a bolus injection of physiological saline (125 mM NaCl, 2.5 mM KCl, 2 mM CaCl_2_, 1 mM MgSO_4_, 1 mM NaH_2_PO_4_, 10 mM HEPES, and 3 mM glucose at a pH of 7.7 with NaOH at room temperature) was administered via the jugular cannula. The bolus volume was calculated to increase the average stroke volume by fivefold for each particular animal, mimicking the possible increase in venous return associated with breath hold, ventilation, or movement (6, 60). Based on existing data suggesting total blood volume is ∼7% of total body mass in this species (33), and under the assumption that 1 g body mass is equal to 1 ml blood volume, the average volume load was 2.2 ± 0.4% of total blood volume. The immediate change in pressure and flows were recorded during this period (∼1 min) and then during the following sustained elevated increase in pressure and flows (∼5 min). The animal was then left for ∼25 min for pressure and flows to return to preinjection values, termed the recovery period, before a final 5-min recording was taken (Fig. 1*B*). After completion of experimental protocol, all cannulas and flow probes were removed, the animals were euthanized by intravenous administration of sodium pentobarbital (150 mg/kg), and the heart was excised.

#### Ex vivo ventricular passive pressure-volume curves.

The intact isolated heart (free from cannulas) was washed in phosphate-buffered saline and placed into an organ bath containing Ringer solution (125 mM NaCl, 2.5 mM KCl, 2 mM CaCl_2_, 1 mM MgSO_4_, 1 mM NaH_2_PO_4_, 10 mM HEPES, and 3 mM glucose at a pH of 7.7 with NaOH at room temperature) at 25 ± 0.3°C to which 60 mM 2,3 butanedione monoxime (BDM) was added to prevent active cross-bridge cycling. Pressure-volume curves from ventricles of both the cold-acclimated group and control group were generated at the same common control temperature, of 25 ± 1°C, to isolate the effects of chronic remodeling on myocardial stiffness from the acute effects of temperature. A cannula containing 25 ± 0.3°C Ringer solution with BDM was fed through the left aorta into the ventricular lumen and secured, using 0-0 silk thread (Harvard Apparatus), occluding the vessel. A second cannula, containing saline solution (0.9% NaCl), was connected to a pressure transducer and fed through the common pulmonary artery into the ventricle lumen. Again the cannula was secured in place with 0-0 silk thread, occluding the vessel. The pressure transducer was calibrated daily against a static water column and recorded at 50 Hz using a PowerLab 16/35 data recording system (ADInstruments) and LabChart Pro software (v 7.2.5; ADInstruments). All other inflow and outflow vessels were occluded, using 0-0 silk thread, making the ventricle a sealed chamber with the two cannulas inside. With the use of a calibrated precision syringe pump (INFORS), Ringer solution with BDM was pumped into the ventricle at 0.05 ml/min until maximum volume was achieved, determined by a drop in the pressure trace following the protocol of Keen et al. (39). All passive filling experiments were conducted <9 h after the original injection of sodium pentobarbital. The two atria and the ventricle were separated, their mass was determined to the nearest milligram, and they were fixed in 4% paraformaldehyde solution before being processed and embedded in paraffin wax.

#### Tissue histology.

Fibrillar collagen and elastin content were analyzed semiquantitatively following the methodology of Keen et al. (39). Briefly, Formalin-fixed tissue samples were processed, embedded in paraffin wax, sectioned at 5 μm (Leica RM2255 microtome; Leica, Wetzlar, Germany), and mounted onto glass slides (Super frost plus; Thermo Fisher Scientific, Waltham, MA). Serial sections from each sample were stained with picro-sirus red for collagen (37) and Miller's elastic stain for elastin (48). Picro-sirus red images were quantified using polarized light microscopy and Miller's elastic images were quantified using bright-field microscopy. Mean fibrillar collagen and elastin contents were expressed as a percentage of total tissue cross-sectional area, excluding the epicardial surface, determined using ImageJ (57). All histological analysis was conducted blind to the acclimation group. Three tissue sections were considered for each individual to ensure consistency in measurements. On each tissue section three separate image montages were taken along transects across the full diameter of the tissue cross section.

#### Calculations and statistics.

Pulmonary blood flow (Q_pul_) was determined directly based on the flow probe output, while total systemic blood flow (Q_sys_) was calculated as the sum of the blood flow recorded from the left aorta, left carotid and subclavian arteries, and the right bundle. Total blood flow (Q_total_) was the sum of Q_pul_ and Q_sys_. Net cardiac shunt flow (Q_shunt_) was calculated as the difference between Q_pul_ and Q_sys_ (Q_pul_ − Q_sys_); therefore, a positive Q_shunt_ indicates a left to right (L-R; net pulmonary) shunt and a negative value indicates a right to left (R-L; net systemic) shunt (12, 43, 69). All flow data were standardized to body mass (kg). Heart rate (*f*_H_) was calculated on the basis of the instantaneous blood flow profile in the left aorta. Total stroke volume (VS_tot_) was calculated as Q_total_/*f*_H_ and standardized to body mass (kg). Systemic resistance (R_sys_) was calculated as mean systemic blood pressure relative to systemic blood flow (P_sys_/Q_sys_), under the assumption that atrial pressure is zero, and standardized to body mass (kg). Ventricular contractility (dP/d*t*) was calculated as the maximum rate of pressure increase over six heartbeats, taken from the cannula positioned in the cavum arteriosum.

For all in vivo recordings, and calculations based on in vivo recordings, significant differences between acclimation temperatures and within groups were assessed by two-way repeated measures ANOVA/General Linear Model (GLM), with the cardiovascular parameter as the dependent variable, and stage of experiment and acclimation group as the fixed factors. When significance was found, a Sidak multiple comparisons post hoc test was conducted to assess significance between acclimation groups at each stage of the experiment. Differences between means within groups were subsequently assessed by Tukey's multiple comparisons post hoc test. Mass parameters and differences in collagen and elastin deposition were analyzed separately by multiple unpaired *t*-tests for parametric data or Mann Whitney *U*-tests for nonparametric data, with each parameter as the test variables and acclimation group as the grouping variable. Each of these statistical analyses were performed using Prism v6.04 (GraphPad Software, La Jolla, CA).

Chamber filling volume was calculated from filling time by the equation:

$$\text{v}\text{o}\text{lu}\text{me}\;(\text{ml})=\text{time}\;(\text{s})\times \frac{0.05}{60}$$

The effect of temperature acclimation on the pressure-volume relationship was assessed by ANCOVA with pressure as the dependent variable, volume and acclimation group as fixed factors, and body mass as the covariate, with a Tukey post hoc test for differences between groups using R (53). For all analyses, significance was considered at *P* < 0.05. Values are presented as mean ± SE throughout unless otherwise stated. Statistical details for each measurement are given in the figure legends.

## RESULTS

### 

#### Heart and chamber mass.

Body mass (1,438.1 ± 115.4 vs. 1,266.4 ± 80.5 g), heart mass (3.07 ± 0.24 vs. 2.84 ± 0.211 g), left atrial mass (0.26 ± 0.034 vs. 0.24 ± 0.034 g), right atrial mass (0.40 ± 0.035 vs. 0.39 ± 0.035 g), ventricular mass (2.24 ± 0.17 vs. 2.07 ± 0.14 g), relative heart mass (0.22 ± 0.013 vs. 0.22 ± 0.009 g), and relative ventricular mass (0.16 ± 0.007 vs. 0.16 ± 0.007 g) were not different between cold-acclimated and control animals, respectively.

#### Effects of thermal acclimation on baseline in vivo cardiac function.

**Fig. 2.**
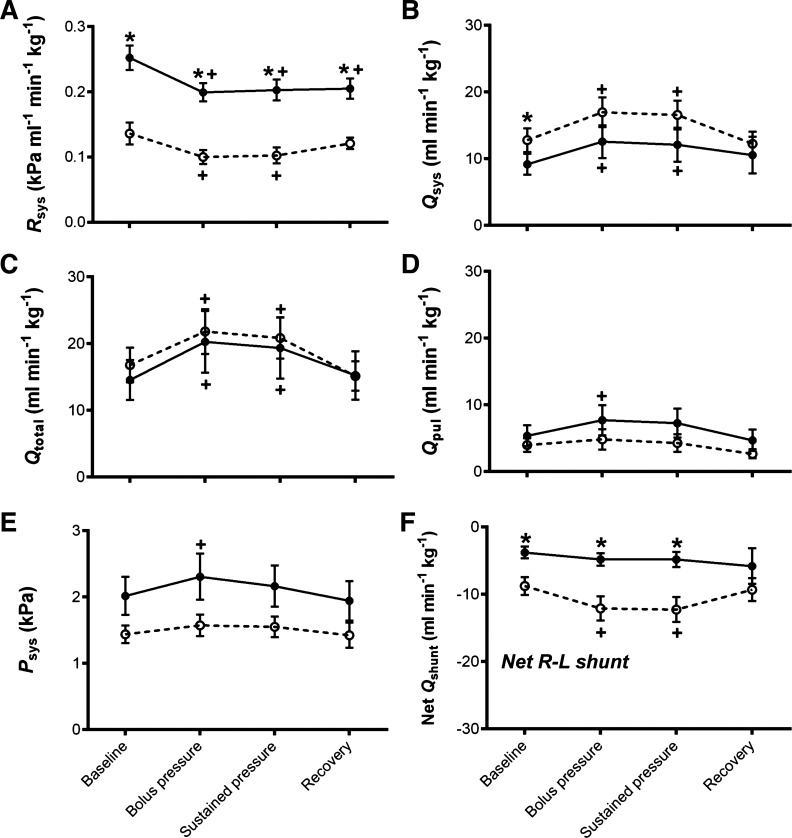
Blood pressures and flows. In vivo measurements of systemic resistance (*R*_sys_; *A*), systemic blood flow (Q_sys_; *B*), pulmonary blood flow (Q_pul_; *C*), total cardiac output (Q_total_; *D*), systemic pressure (*P*_sys_; *E*), and net cardiac shunt flow (Q_shunt_ = Q_pul_ − Q_sys_; *F*) in cold-acclimated (open circles, dashed line) and control (closed circles, solid line), anesthetized and artificially ventilated, freshwater turtles assessed at 25°C. Values are displayed as means ± SE; *n* = 10. Flow values have been standardized to mass for graphical representation. *P < 0.05, significant differences between acclimation groups; +*P* < 0.05, significant changes from the baseline value within groups during the experiment [general linear model (GLM)].

**Fig. 3.**
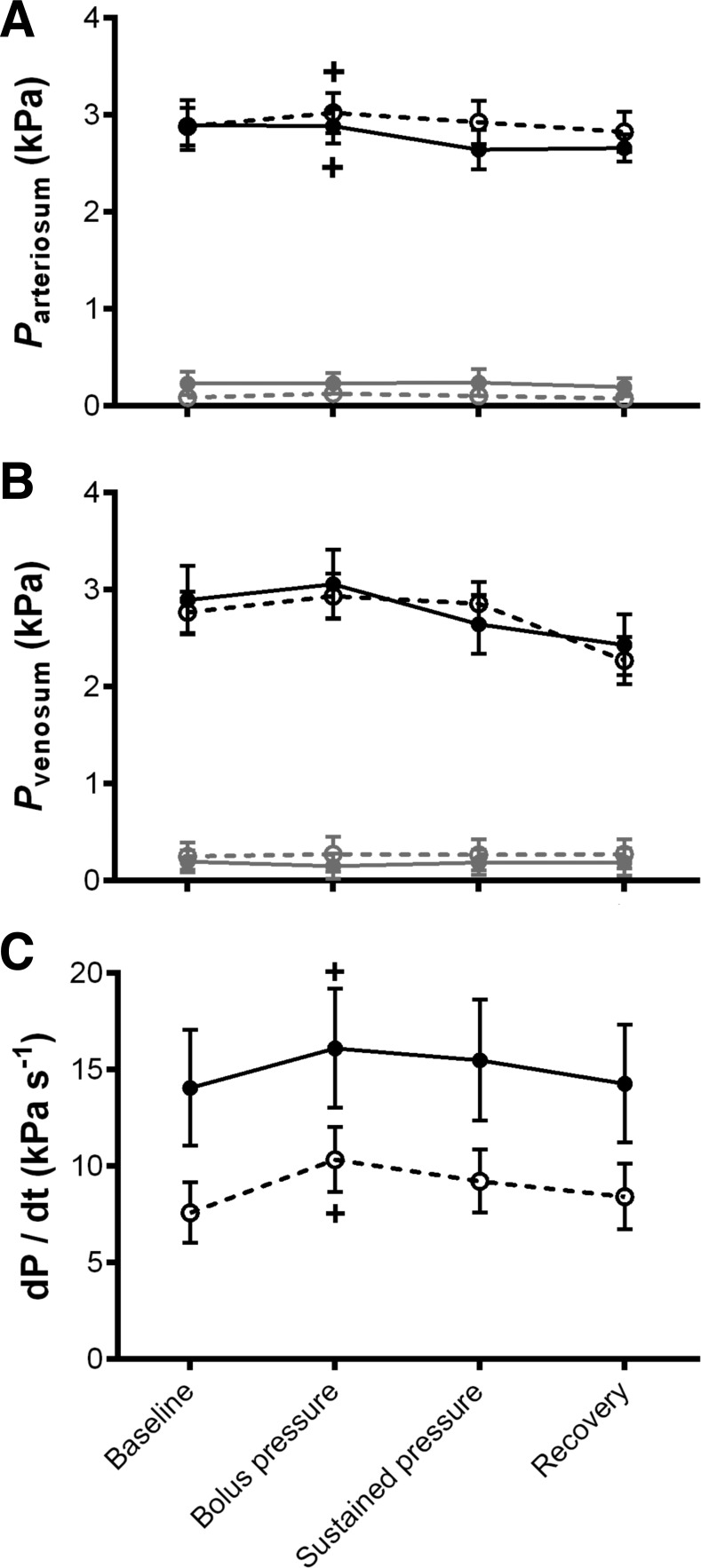
Intraventricular pressure and contractility. In vivo measurements of maximum (black) and minimum (grey) pressure in the cavum arteriousm (*P*_arteriosum_; *A*) and the cavum venosum (*P*_venosum_; *B*), and ventricular contractility (dP/d*t*; *C*) in cold-acclimated (open circles, dashed line) and control (closed circles, solid line), anesthetized and artificially ventilated, freshwater turtles assessed at 25°C. Values are displayed as means ± SE; *n* = 10. +*P* < 0.05, significant changes from the baseline value within groups during the experiment (GLM).

**Fig. 4.**
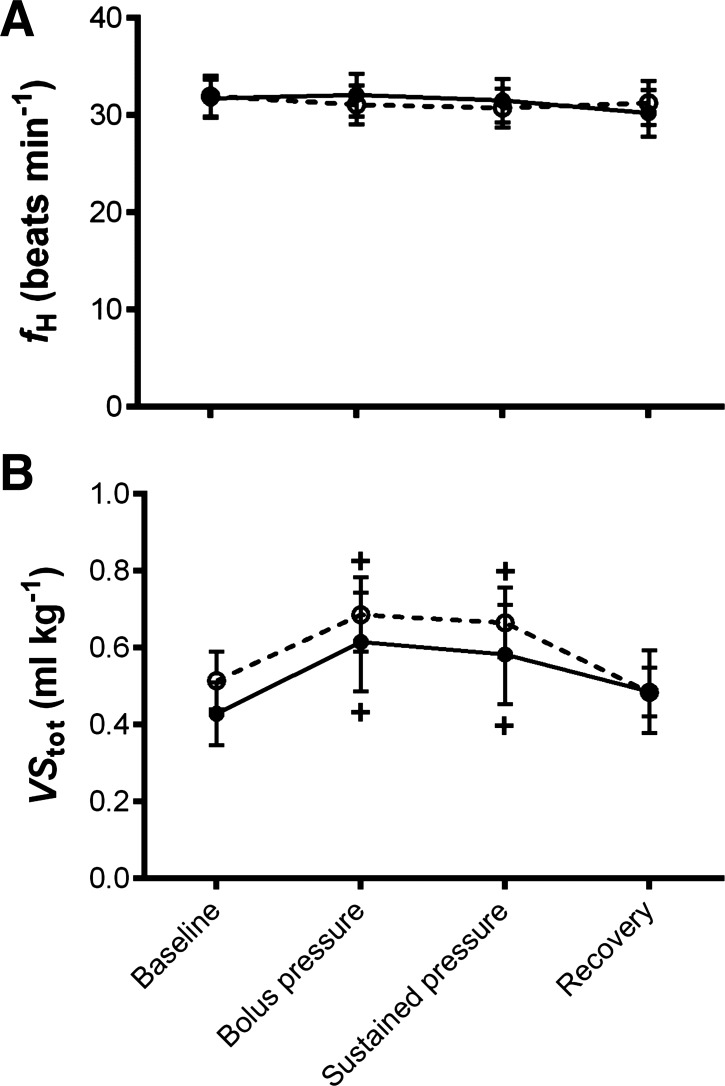
Cardiac performance. In vivo measurements of heart rate (*f*_H_; *A*) and total stroke volume (VS_tot_; *B*) in cold-acclimated (open circles, dashed line, striped bar) and control (closed circles, solid line, solid bar), anesthetized and artificially ventilated, freshwater turtles assessed at 25°C. Values are displayed as means ± SE; *n* = 10. VS_tot_ has been standardized to mass for graphical representation. +*P* < 0.05, significant changes from the baseline value within groups during the experiment (GLM).

Cold acclimation resulted in a 1.8-fold reduction in systemic resistance (R_sys_) compared with control animals [*R*^2^ = 0.67, *F*_(1,53)_ = 79.25, *P* < 0.0001; Fig. 2*A*]. This reduction in R_sys_ contributed to a 1.4-fold increase in systemic blood flow (Q_sys_) in cold-acclimated compared with control animals [*R*^2^ = 0.38, *F*_(1,58)_ = 8.69, *P* < 0.005; Fig. 2*B*]. As there was no effect of temperature acclimation on total cardiac output (Q_total_; Fig. 2*C*), pulmonary flow (Q_pul_; Fig. 2*D*) or systemic pressure (P_sys_; Fig. 2*E*), both groups had a right to left (R-L; net systemic) cardiac shunt (Q_shunt_; Fig. 2*F*). Cardiac shunting occurs in turtles because they have incomplete ventricular separation of venous and arterial circulations, allowing blood flow to bypass or partially bypass either the systemic or pulmonary circulation. These intracardiac shunt flow (Q_shunt_) patterns, i.e., the direction of the blood shunt, are largely determined by vascular resistance of the systemic and pulmonary circulations (31, 50, 58). However, the combined effect of the greater Q_sys_ and reduction in R_sys_ of the cold-acclimated turtles produced a 2.3-fold higher R-L Q_shunt_ [*R*^2^ = 0.62, *F*_(1,58)_ = 20.69, *P* < 0.0001] and a 1.8-fold greater shunt fraction compared with control [*R*^2^ = 0.31, *F*_(1,58)_ = 7.02, *P* < 0.05]. Interestingly, despite changes in blood flow distribution, we did not find an effect of temperature acclimation on maximum or minimum intraventricular pressure between two of the cava in the heart (P_arteriosum_ and P_venosum_; Fig. 3, *A* and *B*) nor was there a change in ventricular contractility (Fig. 3*C*). Finally, there was no difference between resting heart rate (*f*_H_) or total stroke volume (VS_tot_) values between cold-acclimated and control animals (Fig. 4, *A* and *B*).

#### In vivo cardiovascular response to volume load.

The bolus injection, designed to give a fivefold increase in venous return volume, increased VS_tot_ in both groups, which remained elevated during the sustained pressure period {for cold [*R*^2^ = 0.75, *F*_(3,18)_ = 18.08, *P* < 0.0001] and for control [*R*^2^ = 0.42, *F*_(3,18)_ = 4.32, *P* < 0.05]; Fig. 4*B*}. This increase in VS_tot_ directly elevated Q_total_ in both groups during the bolus injection and sustained pressure period {for cold [*R*^2^ = 0.72, *F*_(3,18)_ = 15.39, *P* < 0.0001] and for control [*R*^2^ = 0.37, *F*_(3,18)_ = 3.57, *P* < 0.05]; Fig. 2*C*}. The *f*_H_ was not affected by the volume load (Fig. 4*A*). The increase in Q_total_ translated into an increase in Q_sys_ in both temperature groups {for cold [*R*^2^ = 0.72, *F*_(3,18)_ = 15.48, *P* < 0.0001] and for control [*R*^2^ = 0.37, *F*_(3,18)_ = 3.57, *P* < 0.0001]; Fig. 2*B*} and Q_pul_ in control animals only [*R*^2^ = 0.39, *F*_(3,18)_ = 4.48, *P* < 0.05; Fig. 2*D*], reducing the net R-L shunt in cold-acclimated animals [*R*^2^ = 0.52, *F*_(3,18)_ = 6.58, *P* < 0.005; Fig. 2*F*]. The bolus injection caused a decrease in *R*_sys_ in both groups {for cold [*R*^2^ = 0.55, *F*_(3,18)_ = 7.38, *P* < 0.005] and for control [*R*^2^ = 0.68, *F*_(3,18)_ = 12.93, *P* < 0.0001]; Fig. 2*A*}, but an increase in *P*_sys_ in the control group only [*R*^2^ = 0.42, *F*_(3,18)_ = 4.38, *P* < 0.05; Fig. 2*E*]. Maximum *P*_arteriosum_ was increased in the cold-acclimated animals; however, this was decreased in the control animals {for cold [*R*^2^ = 0.50, *F*_(3,18)_ = 6.134, *P* < 0.05] and for control [*R*^2^ = 0.54, *F*_(3,18)_ = 8.31, *P* < 0.05]; Fig. 3*A*}. Following the bolus injection there was an increase in ventricular contractility in both groups {for cold [*R*^2^ = 0.48, *F*_(3,21)_ = 6.58, *P* < 0.05] and for control [*R*^2^ = 0.73, *F*_(3,24)_ = 22.09, *P* < 0.001]; Fig. 3*C*}. After the recovery period, *R*_sys_ remained elevated in the control group whereas it returned to baseline levels in the cold-acclimated (Fig. 2*A*).

#### Effects of thermal acclimation on the in vivo cardiovascular response to volume load.

Following the bolus injection, R_sys_ remained twofold higher in control compared with cold-acclimated animals during both the bolus injection and the sustained pressure phase (*P* < 0.05; Fig. 2*A*). During both the bolus injection and sustained pressure phase Q_sys_ was slightly increased, while Q_pul_ and P_sys_ were slightly decreased by cold acclimation compared with control (Fig. 2, *B*, *D*, and *E*). The R-L Q_shunt_ persisted in both acclimation groups, however, the reduced R_sys_, Q_pul_, and P_sys_ combined with the increased Q_sys_ meant the R-L Q_shunt_ remained more pronounced (Fig. 2*F*), with a greater shunt fraction, in the cold-acclimation compared with the control animals during both the bolus injection and sustained pressure phase (*P* < 0.05). Temperature acclimation did not influence Q_total,_ P_arteriosum_, P_venosum_, *f*_H_, or VS_tot_ during either the bolus injection or sustained pressure phase (Figs. 2*C*, 3, and 4).

#### Thermal remodeling of ex vivo ventricular compliance.

**Fig. 5.**
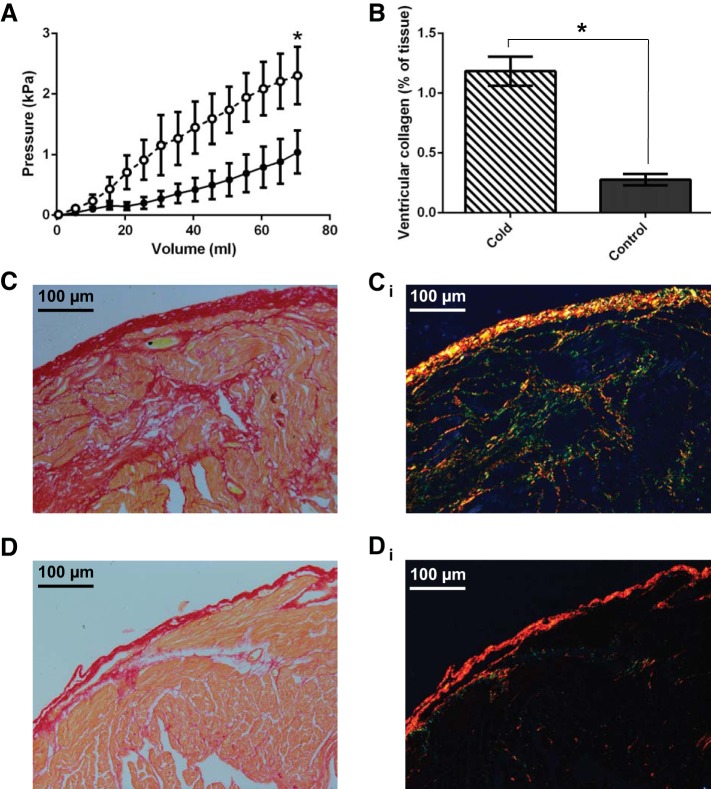
Ventricular stiffness and connective tissue content. Ex vivo ventricular passive pressure-volume relationships (*A*) and semiquantitative analysis of ventricular collagen content (*B*) in cold-acclimated (open circles, dashed line) and control (closed circles, solid line) freshwater turtles. Representative micrographs of picro-sirus red stained ventricular tissue, for cold-acclimated freshwater turtles viewed under bright-field (*C*) and plane polarized light (*C*_*i*_) and control freshwater turtles viewed under bright-field (*D*) and plane polarized light (*D*_*i*_). Values are displayed as means ± SE; *n* = 10. Pressure has been standardized to start at 0 kPa for graphical representation. *P < 0.05, significant differences between acclimation groups (GLM).

The maximum *P*_arteriosum_ following the volume load was increased in the cold-acclimated and reduced in the control group, compared with baseline values. The increased pressure in response to volume suggests a decrease in ventricular compliance with cold acclimation. To assess the functional effects of cardiac remodeling on the passive properties of the thermally acclimated ventricle we generated ex vivo passive filling curves from freshly isolated intact ventricles treated with BDM at a common test temperature of 25 ± 0.3°C. Thermal acclimation altered the pressure-volume relationship during filling [*R*^2^ = 0.75, *F*_(7,379916)_ = 167200, *P* < 0.001] revealing increased stiffness in the cold compared with controls (Fig. 5*A*).

#### Thermal remodeling of connective tissue.

Increased myocardial stiffness can be due to a remodeling of the extracellular matrix in both mammals and ectotherms (8, 39, 42). We used picro-sirus red to determine fibrillar collagen deposition in the turtle ventricle and major outflow vessels. Ventricular collagen content was 4.3-fold higher in cold-acclimated animals compared with controls (*R*^2^ = 0.69, *P* < 0.0001; Fig. 5*B*), as shown by the higher degree of dark red staining, when visualized under bright-field light, and the increased number of birefringent fibers, when visualized under plane polarized light (Fig. 5, *C*, *C*_*i*_, *D*, and *D*_*i*_). We did not detect elastin in the turtle ventricular myocardium except in coronary vessels (not shown).

**Fig. 6.**
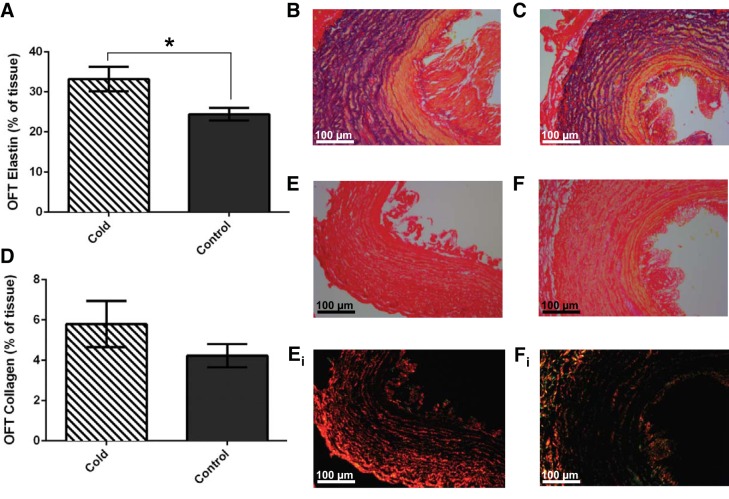
Connective tissue content of the major outflow vessels. *A*: semiquantitative analysis of elastin content in the major outflow (OTF) arteries for cold-acclimated (striped bar) and control (solid bar) freshwater turtles. This result is shown by representative micrographs of a Miller's elastic stained cold-acclimated (*B*) and control vessel wall (*C*). *D*: semiquantitative analysis of collagen content in the major outflow arteries for cold-acclimated (striped bar) and control (solid bar) freshwater turtles. This result is shown by representative micrographs of a picro-sirus red stained cold-acclimated tissue under bright-field (*E*) and plane polarized light (*E*_*i*_) and control tissue under bright-field (*F*) and plane polarized light (*F*_*i*_). Values are displayed as means ± SE; *n* = 10. *P < 0.05, significant differences between acclimation groups (GLM).

The major outflow vessels contain a thick layer of connective tissue that provides structural support and elastic recoil. With high cardiac preload and VS_tot_ associated with cold acclimation, the balance of collagen and elastin in these vessels may be critical to vascular function (4, 24, 52). Staining the vessels with Miller's elastic stain revealed a 1.4-fold increase in elastin content in the artery wall following cold acclimation (*R*^2^ = 0.51, *P* < 0.05; Fig. 6*A*), which is visualized by the increased black staining of the elastic lamellae following cold acclimation compared with controls (Fig. 6, *B* and *C*, respectively). We were unable to statistically resolve differences between the acclimation groups for fibrillar collagen in the major outflow arteries (Fig. 6*D*). Representative bright-field images with their corresponding plane polarized light image are shown following cold acclimation in Fig. 6, *E* and *E*_*i*_, respectively, and for controls in Fig. 6, *F* and *F*_*i*_, respectively.

## DISCUSSION

Ectotherms show a wide range of physiological responses to acute and chronic temperature change. Freshwater turtles endure large fluctuations in seasonal temperature in their native environments (66). These seasonal temperature changes directly affect many physiological processes, with extreme cold triggering winter long hibernation or brumation (66). Here, freshwater slider turtles were exposed to chronic cold (∼5°C) to simulate a winter phenotype. In vivo cardiovascular function, ex vivo ventricular compliance, and connective tissue content of the ventricle and major outflow vessels were assessed. However, unlike previous studies, all experiments were conducted at a common control temperature (25 ± 0.3°C) to differentiate thermal remodeling of the cardiovascular system from the direct effects of temperature. Our findings indicate cold acclimation increased R-L Q_shunt_ by a reduction in R_sys_ and an increase in Q_sys_. Furthermore, cold-induced cardiovascular remodeling increased ventricular stiffness during passive filling.

### 

#### Critique of the methods.

The acclimation duration in this study (>8 wk before the start of experiments) was longer than used in a number of previous studies on freshwater turtles (12, 27, 62, 63) but was chosen to agree with the timeframe required for a cardiac remodeling response to temperature in other ectotherms (e.g., the rainbow trout) (39, 42). Second, the majority of previous studies on cold-acclimated turtle heart function were conducted on recovered animals (27, 62, 63). Anesthesia with pentobarbital blunts autonomic tone on the cardiovascular system and is, therefore, useful in assessing hemodynamic effects of central nervous system regulation (13, 36, 50). A number of studies have previously assessed cardiac function while animals remained under anesthesia (Table 1) (10, 11, 13, 21, 31, 36, 50). At warm temperatures anesthesia with sodium pentobarbital has been shown to cause a L-R (net pulmonary) shunt as it blocks the cholinergic mediated constriction of the pulmonary artery that is normally associated with apnoea (51). Third, we also continuously artificially ventilated our animals to prevent hypoxia. Lung ventilation in recovered animals is associated with an increased *f*_H_ and Q_pul_ and a reduction in the overall R-L shunt (58, 69, 71), suggested to be due to vagal and adrenergic tone (27, 30). Using a continuous artificial ventilation protocol has been shown to remove pulmonary CO_2_ more effectively (46) than the episodic ventilation pattern seen in a nonanesthetized and unventilated animal; a series of consecutive breaths interspersed by periods of apneas causing bodily gas stores to fluctuate (22, 46, 58). However, *f*_H_ and overall Q_pul_ are not affected by this continuous ventilation pattern (46). Finally, the turtles used in this study were maintained in water throughout the acclimation period in both groups. To adjust buoyancy turtles may retain water in their urinary bladder or cloacal bursac when maintained in water for prolonged periods of time (34, 55). We did not find any difference in total body mass between control and cold-acclimated turtles, in agreement with previous studies (27, 51). However, as we did not measure body mass before and after acclimation periods we are unsure if it was altered by water retention.

**Table 1.** In vivo cardiac parameters in anesthetized freshwater slider turtles (Trachemys scripta) at warm temperatures (∼25°C)

Q_pul_, ml·min^−1^·kg^−1^	Q_sys_, ml·min^−1^·kg^−1^	*f*_H_, min^−1^	VS_tot_, kg^−1^	R_sys_, kPa·ml^−1^ ·min^−1^·kg^−1^	Q_shunt_, ml·min^−1^·kg^−1^	Reference
∼ 16	∼45	∼26	∼2.7	∼0.05	∼−29	Joyce and Wang (35)
31.9	31.1	20.7	3.1	0.13	−0.23	Galli et al. (21)
60.4	28.0	35.6	2.6	0.14	2.34	Overgaard et al. (50)
27.3	22.2	37.5	1.32	1.72	5.1	Crossley et al. (13)
35.1	31.9	38.5	1.73	0.12	3.2	Crossley et al. (11)
42.3	19.5	43.2	1.37	0.15	22	Hicks et al. (31)
57.4	—	44.0	—	—	—	Hicks and Comeau (30)
33.8	31.9	38.5	1.73	0.12	1.9	Comeau and Hicks (10)
5.4	9.2	31.2	0.43	0.25	−3.2	Present study

Data from previous studies is presented alongside baseline cardiac parameters from the present study for control animals (acclimated at 25°C) assessed at 25°C. Q_pul_, pulmonary blood flow; Q_sys_, systemic blood blow; *f*_H_, heart rate; VS_tot_, total stroke volume; *R*_sys_, systemic resistance; Q_shunt_, net cardiac shunt flow.

#### The effect of thermal acclimation on systemic pressure and blood flow.

Our findings suggest cold acclimation reduced R_sys_, increased Q_sys_, and, therefore, increased R-L Q_shunt_. Previously, cold acclimation has been reported to reduce both P_sys_ and systemic conductance (*G*_sys_; 1/R_sys_) in turtles (12, 27, 61–63). Speculatively, this may be due to atrial natriuretic peptide (ANP), which is present in the testudine heart (65). In mammals and reptiles, ANP is released by cardiomyocytes in response to pressure or volume induced myocardial stretch and has systemic vasodilatory action (41, 49); however, it is unclear if the action of ANP is blunted by cold temperatures like some other endocrine functions (45). Vasodilation is consistent with previous studies that show arterial hypotension following cold acclimation when animals are studied at their acclimation temperatures (27). However, temperature is also inversely related to blood viscosity (44, 54, 56), which leads to higher viscosity at low temperatures and an overall increase in R_sys_ (27, 62, 63). The overall decrease in R_sys_ following cold acclimation, in the current study, suggests either a functional change in the vasculature, changes in vascular physical properties, or reduced viscosity at 25°C. However, Saunders and Patel (56) report very little difference between blood viscosities of red-eared sliders when tested at 25°C, except at very low shear rates, regardless of whether turtles were acclimated to 25 or 5°C. Furthermore, we found an increased Q_sys_ when cold-acclimated animals were studied at 25°C, which differs from the findings previously reported for animals studied at 5°C (62, 63). We also report an increased elastin content found in the major outflow arteries, suggesting increased compliance and elastic recoil in these vessels. Indeed, cold acclimation may induce decreases in R_sys_ via a structural change in vasculature compliance; however, further studies are needed to test this speculation experimentally.

#### Cardiovascular remodeling with temperature acclimation.

In both acclimation groups, a volume load of ∼2.2% of total blood volume increased VS_tot_, P_sys_, and Q_total_ in agreement with previous studies on this species (14). In control animals this caused both Q_pul_ and Q_sys_ to increase; however, in cold-acclimated animals only Q_sys_ increased. This finding could relate to acclimation-induced vasculature remodeling to increase compliance in cold-acclimated animals, reducing the effect of changes in ejected VS_tot_ or Q_total_ on P_sys_ (20). This idea is supported by our histological analysis of the outflow arteries, which suggests an increase in compliant elastin fibres in the cold-acclimated group. Increased vascular compliance would be able to accommodate the large increases in volume as well as reduce afterload pressure and systolic wall stress, potentially allowing systolic pressure and ejection fraction to be preserved (40). The increased elastin content would also improve recoil in the arteries, helping them to more efficiently smooth blood flow and regain their shape faster in diastole (25, 67).

The maximum P_arteriosum_ following volume load was increased in the cold-acclimated and reduced in the control group, compared with baseline values. The increased pressure response to volume suggests a decrease in ventricular chamber compliance with cold acclimation. This finding agrees with the result of our ex vivo pressure-volume relationships and ventricular connective tissue data, which suggest that the cold-acclimated ventricle was stiffer than controls, with a higher deposition of collagen in the myocardial wall. To our knowledge this is the first time pressure-volume curves have been generated in the turtle heart; however, Farrell et al. (16) used an in situ working heart preparation from slider turtles to show increased sensitivity to filling pressure following acute cold exposure, which is consistent with our findings. Data from mammals also suggest that a shift in the Frank-Starling curve due to decreased ventricular compliance can preserve systolic function and increase preload recruitable stroke work (1, 3, 23, 47). Moreover, ventricular stiffness is correlated with increased systolic pressure sensitivity to cardiac preload, and therefore, increases in central blood volume give greater increases in systolic developed pressure, even in the absence of cardiac hypertrophy as shown in these turtles (9, 20). Structural remodeling causing increased ventricular stiffness can also be associated with diastolic dysfunction and increased diastolic pressure (2, 41). It is unclear whether a reduction in cardiac compliance with associated fibrosis is beneficial or maladaptive in the turtle heart following cold acclimation. Diastolic function appears normal at low volumes as minimum P_arteriosum_ and P_venosum_ were not affected by thermal acclimation. We did not find a difference in ventricular contractility following temperature acclimation. We speculate that the lack of change is explained by thermal remodeling in the turtle heart being a physiological, rather than a pathological, response.

Maximum and minimum intraventricular pressures were similar, independent of temperature acclimation, which agrees with previous data on most reptiles (5, 35, 58). It is likely that differential pressure generation is unnecessary due to the low P_sys_ in the turtle and, therefore, low risk of pulmonary edema in the pulmonary vasculature (7, 29). In more active reptile species, such as the Burmese python (*Python molurus*), the systemic side of the heart (or cavum arteriousm) generates higher pressure than the pulmonary side (68). However, in the python heart the muscular ridge between the cava is larger than that in the turtle heart, the ventricular wall surrounding the systemic side of the heart is thicker than the pulmonary side, and there is a greater degree of blood flow separation (17, 68, 70). Interestingly, although there was no change in intraventricular pressure, we did see changes in cardiac shunting patterns with an increased R-L Q_shunt_ following cold acclimation. This finding suggests that cardiac shunting is controlled entirely by peripheral resistance rather than modulating pumping pressure within the ventricle.

#### The effect of thermal acclimation on heart rate, stroke volume, and cardiac output.

Previous studies report decreased *f*_H_ and Q_total_, with a corresponding increase in VS_tot_ following cold acclimation (15, 62–64). However, we did not find differences in these parameters between temperature groups. Indeed, our values for these parameters in both groups better correlate to previously reported baseline levels under anesthesia at ∼25°C (Table 1) (10, 11, 13, 21, 30, 31, 36, 50). Our blood flow values are lower than that of most other studies. The basis for this difference was not clear but may relate to intraventricular pressure cannula placement, which is the main difference between this study and those previously conducted. As the animals in the present study were anesthetized and ventilated, *f*_H_ and Q_total_ are likely higher than if the animals were nonanesthetized and apnoeic as anesthesia blunts vagal tone on the heart and pulmonary artery (11, 50). Our findings suggest that, although cold-acclimated animals show depressed *f*_H_ and Q_total_ with an increased VS_tot_ when studied at 5°C, the main driver of these changes is acute cold rather than cold acclimation per se. Indeed, Stecyk et al. (63) came to a similar conclusion in regard to *f*_H_ while working with spontaneously beating whole heart preparations.

### Perspectives and Significance

Prolonged cold initiates profound alterations in ectotherm physiology. In the case of the freshwater turtle, acute cold triggers large depressions in metabolic rate to initiate winter hibernation/brumation and results in modifications in cardiovascular function (26). The data presented in this study builds from previous studies in freshwater turtles (15, 27, 28), indicating that cold acclimation alters cardiac shunting patterns with an increased R-L Q_shunt_, achieved through a reduction in R_sys_ and an increase in Q_sys_. Furthermore, cold acclimation increased the heart's sensitivity to an in vivo volume load, which may be relevant during hibernation, when diving turtles have been suggested to increase VS_tot_ by up to fivefold (6). Ex vivo passive filling of the ventricle revealed a reduction in ventricular compliance, which was associated with fibrosis of the myocardium. In turn, the major outflow arteries exhibited an increase in elastin content of the elastic lamellae suggesting increased outflow vessel compliance following cold acclimation. These findings suggest that cold-induced structural cardiovascular remodeling alters the hemodynamics of freshwater turtles to limit the stress of high blood preload on the heart. It is possible that these structural changes may also compensate for decreased systolic function associated with low temperatures.

## GRANTS

A. N. Keen was funded by the Biotechonology and Biological Sciences Research Council and this research trip was funded by travel awards from the Fisheries Society of the British Isles and the Royal Society of Biology. D. A. Crossley, 2nd was funded by National Science Foundation CAREER Award IBN IOS-0845741. H. A. Shiels was supported by a Wellcome Trust ISSF Collaborative Research Visit grant.

## DISCLOSURES

No conflicts of interest, financial or otherwise, are declared by the author(s).

## AUTHOR CONTRIBUTIONS

A.N.K. and H.A.S. conception and design of research; A.N.K. and D.A.C.I. performed experiments; A.N.K. analyzed data; A.N.K., H.A.S., and D.A.C.I. interpreted results of experiments; A.N.K. prepared figures; A.N.K. drafted manuscript; A.N.K., H.A.S., and D.A.C.I. edited and revised manuscript; A.N.K., H.A.S., and D.A.C.I. approved final version of manuscript.
